# Mature Cystic Teratoma of the Right Adrenal Gland in a Pediatric Patient: A Case Report

**DOI:** 10.1002/ccr3.71780

**Published:** 2025-12-29

**Authors:** Amit Gautam, Pukar Adhikari, Shishir Bhandari, Jabir Ahamad Miya, Nikesh Tiwari

**Affiliations:** ^1^ Department of Radiology Chitwan Medical College Teaching Hospital Chitwan Nepal; ^2^ Department of Internal Medicine Chitwan Medical College Teaching Hospital Bharatpur Nepal; ^3^ Medical Officer, Himalayan Heart and Healing Bharatpur Nepal

**Keywords:** adrenal teratoma, female, mature cystic teratoma, pediatric, retroperitoneal tumor, suprarenal mass

## Abstract

Adrenal teratomas are rare neoplasms, most often benign but with potential for malignant transformation, particularly in the pediatric population. A seven‐year‐old girl from kawasoti, Nepal, presented with intermittent right‐sided abdominal pain for 2 months, initially attributed to dietary factors such as junk food intake. Ultrasonography revealed a large right suprarenal mass measuring 10.4 × 8.2 cm with internal calcifications. Contrast‐enhanced computed tomography demonstrated a heterogeneous lobulated lesion containing fat, fluid, and calcifications in the right suprarenal region, displacing adjacent structures. The mass was surgically excised via exploratory laparotomy with complete excision of the right‐sided retroperitoneal adrenal mass. Intraoperative findings revealed an 11 × 8 cm firm, encapsulated mass with dense adhesions to the inferior vena cava. Histopathological examination confirmed the diagnosis of mature cystic teratoma. The patient's postoperative course was complicated by wound dehiscence requiring secondary suturing, with subsequent development of a hypertrophic scar managed with intralesional triamcinolone injection. At six‐month follow‐up, the patient remained asymptomatic with no evidence of recurrence. This case highlights the diagnostic challenges of adrenal teratomas in children and emphasizes the importance of considering this rare entity in the differential diagnosis of suprarenal masses. Complete surgical excision remains the definitive treatment with excellent prognosis when the tumor is benign.

## Key Clinical Message

Consider adrenal teratoma in children with large suprarenal masses when imaging demonstrates fat, fluid, and calcific components; complete surgical excision is diagnostic and curative for mature lesions, and long‐term imaging follow‐up is recommended.

**Abbreviations**
AFP: Alpha‐fetoprotein
CECT: Contrast‐enhanced computed tomography
CT: Computed tomography
INR: International normalized ratio
IVC: Inferior vena cava
MCT: Mature Cystic Teratoma
MRI: Magnetic resonance imaging
US/USG: Ultrasonography
VMA: Vanillylmandelic acid

## 1 Introduction

Teratomas are germ cell tumors composed of tissues derived from all three embryonic germ layers: ectoderm, mesoderm, and endoderm [1]. While these tumors commonly occur in gonadal locations, extragonadal teratomas account for only 15% of all teratomas, with the sacrococcygeal region being the most frequent extragonadal site [2]. Primary adrenal teratomas are exceptionally rare, particularly in the pediatric population, with an estimated incidence reported as 0.13% in an institutional series [2, 3].

The retroperitoneum represents an unusual location for teratomas, accounting for approximately 1%–11% of all primary retroperitoneal tumors in children [1, 4]. Among retroperitoneal teratomas, those arising from the adrenal gland are extraordinarily uncommon, with very few pediatric cases reported in the literature since the first pediatric description by Engel et al. in 1968 [5], preceding later reports such as Lam and Lo in 1999 [6]. The rarity of this condition poses significant diagnostic and therapeutic challenges, as clinical presentation is often nonspecific and imaging findings may overlap with more common adrenal masses such as neuroblastoma, pheochromocytoma, or adrenocortical carcinoma [7].

The pathogenesis of adrenal teratomas remains unclear, with several theories proposed including aberrant migration of primordial germ cells during embryogenesis, metaplasia of adrenal tissue, or development from displaced embryonic rests [8]. Most adrenal teratomas are benign mature cystic lesions, though malignant transformation has been reported but is rare; published rates vary by tumor site (ovarian MCT ~1%–3%); adrenal cases of malignant transformation are reported mainly as case reports [9, 10].

Clinical presentation of adrenal teratomas is variable and depends on tumor size, location, and potential hormone production. Most patients present with nonspecific symptoms such as abdominal pain, palpable mass, or are diagnosed incidentally during imaging for unrelated conditions [11]. Laboratory investigations typically reveal normal adrenal hormone levels, distinguishing these lesions from functional adrenal tumors [12].

We present a rare case of a mature cystic teratoma arising from the right adrenal gland in a seven‐year‐old girl, successfully managed with complete surgical excision. This case report aims to contribute to the limited literature on pediatric adrenal teratomas and highlight the diagnostic approach and surgical management of this rare entity.

## 2 Case Presentation

### 2.1 Clinical History

A seven‐year‐old previously healthy girl presented to our tertiary care center with a two‐month history of intermittent right‐sided abdominal pain. The pain was initially attributed to dietary indiscretion such as frequent fast‐food consumption and managed symptomatically with over‐the‐counter analgesics at local pharmacies. The persistent nature of symptoms, associated with school refusal behavior, prompted evaluation at a peripheral hospital where ultrasonography revealed a right suprarenal mass. The patient was subsequently referred to our institution for further management.

The patient's mother reported that the abdominal pain was intermittent, localized to the right subcostal region, and associated with loss of appetite. There was no history of fever, weight loss, changes in bowel or bladder habits, chest pain, fearfulness, excessive sweating, headache, loss of consciousness, forehead sweating, abnormal body movements, blood in the urine, increased frequency of urination, rashes, muscle weakness, irritability, or excessive hair growth. The patient's mother denied any history of trauma to the abdomen.

### 2.2 Past Medical and Family History

The patient had experienced occasional episodes of abdominal pain in the past, managed with medications from local pharmacies. Her birth history revealed a full‐term vaginal delivery with a birth weight of 3900 g, immediate cry after birth, and no neonatal intensive care unit admission. Immunizations were completed according to national guidelines. There was no family history of benign or malignant tumors, endocrine disorders, or unexplained deaths. The family belonged to middle socioeconomic status.

### 2.3 Physical Examination

On examination, the patient appeared alert and well‐nourished. Vital signs were: pulse rate 87 beats per minute, blood pressure 96/70 mmHg, respiratory rate 21 breaths per minute, and afebrile. There was no pallor, icterus, cyanosis, edema, or palpable lymphadenopathy.

Abdominal examination revealed a flat abdomen with a centrally placed umbilicus. A firm, non‐tender mass measuring approximately 4.5 × 8 cm was palpable in the right subcostal region with no associated tenderness in the renal angle or bimanual palpability. The mass moved with respiration, and fingers could not be insinuated between the mass and the costal margin. The mass disappeared on leg raise test, suggesting its intraabdominal location. There was no evidence of ascites. Bowel sounds were present and normal.

### 2.4 Investigations

Initial ultrasonography performed at the referring hospital (October 23, 2024) demonstrated a large, well‐defined heterogeneous mass lesion measuring 10.4 × 8.2 cm in the right suprarenal region with internal echogenic and hypoechoic areas. Discrete areas of calcification were noted within the lesion. The mass was compressing and displacing the right lobe of liver superiorly, causing compression of the inferior vena cava medially, and abutting the superior pole of the right kidney inferiorly. No detectable vascularity was noted within the lesion, and the mass had not crossed the midline.

**FIGURE 1:**
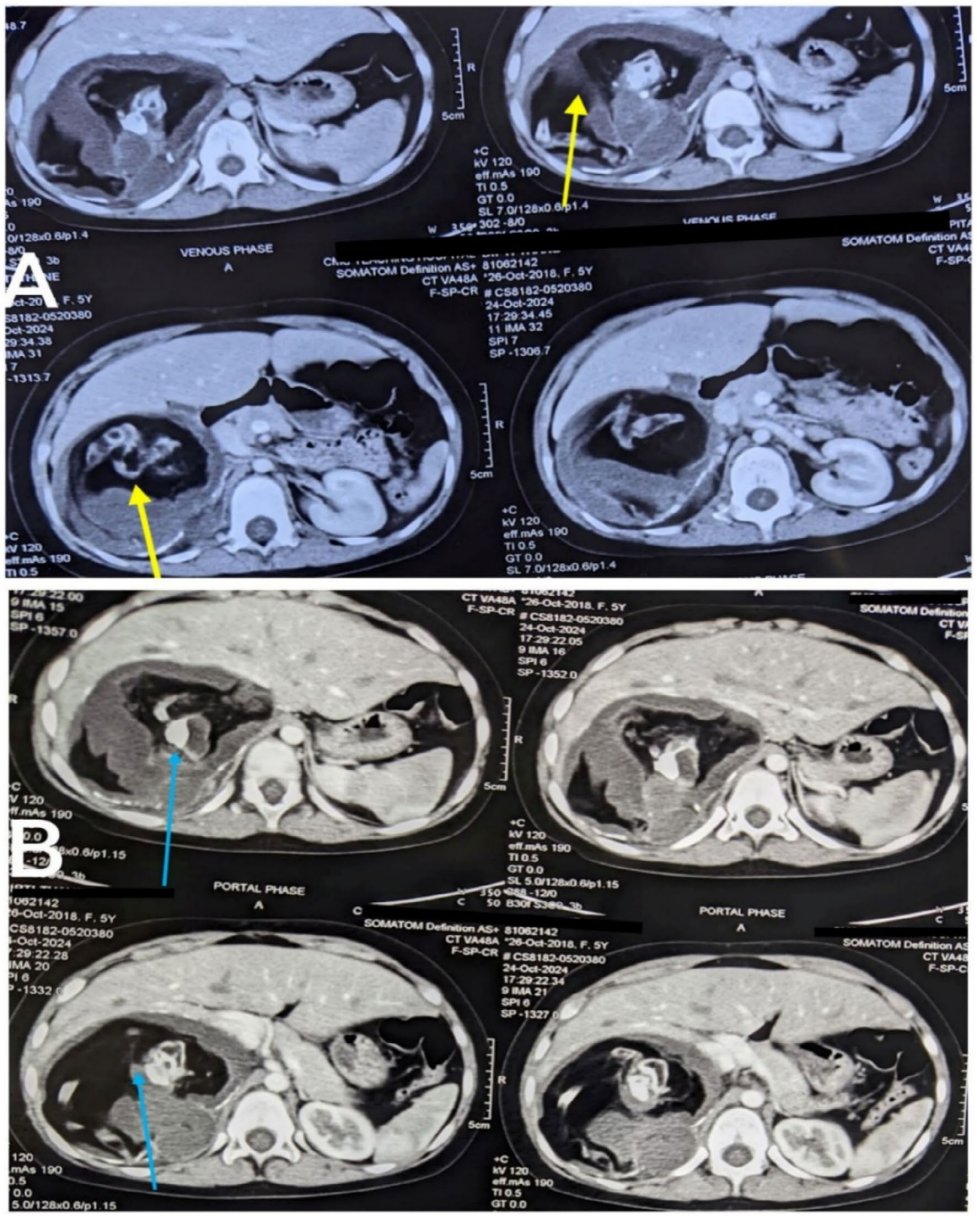
Selected CECT of abdomen and pelvis in axial section showing large right suprarenal lesion with fat, fluid and calcific density (A: Shown by yellow arrow and B: Shown by blue arrow); (A) Shows venous phase, (B) Shows Portal phase.

**FIGURE 2:**
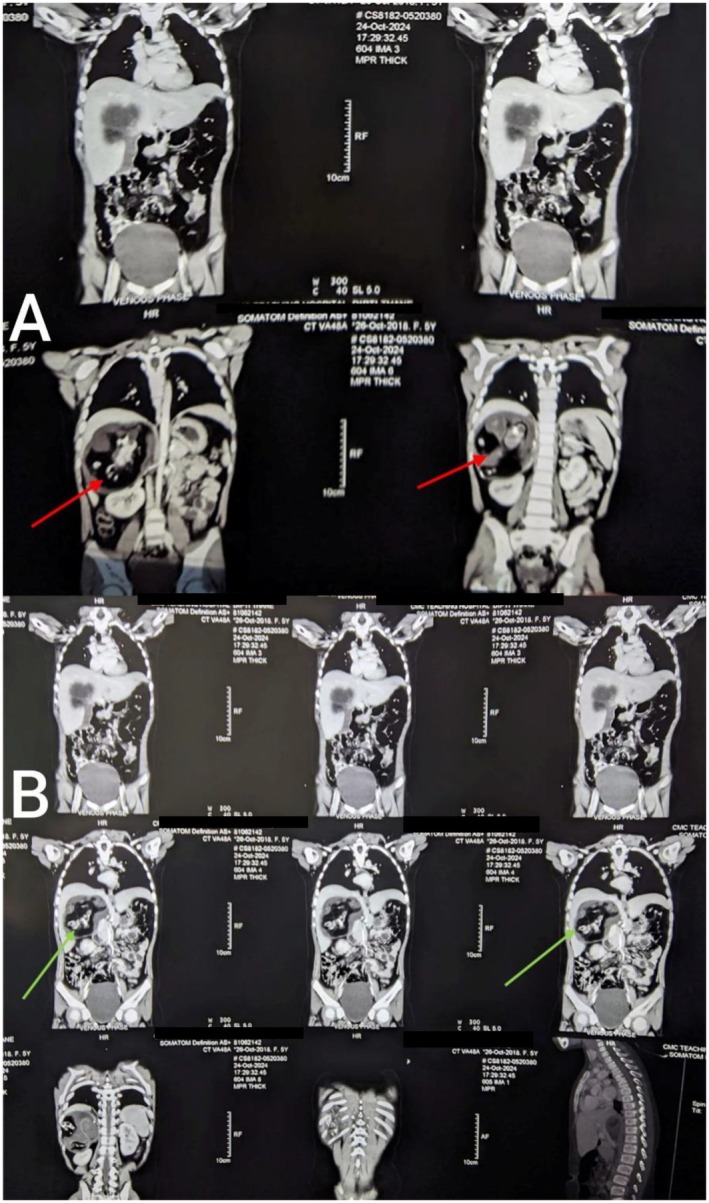
Selected CECT of abdomen and pelvis in coronal section showing large right suprarenal lesion with fat, fluid and calcific density (A: Shown by red arrow and B: Shown by green arrow).

Contrast‐enhanced computed tomography (CECT) of the abdomen and pelvis (October 24, 2024) revealed (Figure 1A,B and Figure 2A,B) a large lobulated heterogeneous lesion with peripheral rim calcifications containing fluid, fat, and calcific densities along with a fat‐fluid level in the right suprarenal region. No enhancement was noted within the lesion in post‐contrast images, with no evidence of midline crossing, vascular encasement, lymphadenopathy, intraspinal extension, ascites, or omental infiltration—features that helped differentiate it from neuroblastoma or other malignant adrenal masses. The right adrenal gland was not separately visualized from the lesion. The mass displaced the right kidney inferiorly and abutted the duodenum, gallbladder, right crus of diaphragm, and segments VI and VII of the right liver lobes. It compressed the intrahepatic portion of the inferior vena cava and was closely related to renal vessels in its medial aspect. The contralateral adrenal gland appeared normal.

Laboratory investigations revealed: alpha‐fetoprotein (AFP) 0.9 ng/mL (normal), complete blood count with hemoglobin 11.3 g/dL, platelet count 238,000/μL (normal), serum albumin 3.9 g/dL, and normal coagulation profile (INR 1.02). Renal function tests were within normal limits. Urinary catecholamines and vanillylmandelic acid (VMA) levels were not assessed as the clinical features didn't suggest otherwise and parents denied any further evaluation.

### 2.5 Treatment

The preoperative diagnosis was suspected adrenal teratoma, with differentials including neuroblastoma and adrenocortical carcinoma ruled out based on imaging and laboratory findings. After initial evaluation and counseling regarding surgical intervention, the family initially deferred surgery due to financial constraints. The patient was managed conservatively with stool softeners and analgesics as needed, with regular follow‐up every 15 days. During this two‐month period, serial ultrasonography showed no significant change in tumor size or characteristics. With persistent symptoms, the family consented to surgical intervention.

The patient underwent transperitoneal exploratory laparotomy with complete excision of the right‐sided retroperitoneal adrenal mass on December 19, 2024, under general anesthesia. An open approach was chosen due to the large tumor size (approximately 11 cm), anticipated adhesions, and limited availability of laparoscopic resources for pediatric patients in our setting. Intraoperative findings included a firm, encapsulated mass measuring 11 × 8 cm located above the right kidney with dense adhesions to adjacent vessels including the inferior vena cava. Multiple small feeding vessels to the mass were identified and ligated, with minimal blood loss (approximately 100 mL). No intraoperative rupture occurred; the specimen was bihalved post‐excision for histopathological examination. The mass was completely excised and sent for histopathological examination. An abdominal drain was placed prophylactically to monitor for any postoperative bleeding or serous collection.

### 2.6 Postoperative Course

The immediate postoperative period was uneventful. The patient was started on a liquid diet on postoperative day one, progressed to soft diet on day two, and normal diet by day three. The abdominal drain placed in the right subcostal region showed minimal output and was removed on postoperative day three. Initial antibiotic therapy consisted of intravenous ceftriaxone for 3 days (prophylactic, given the extensive dissection) followed by oral amoxicillin‐clavulanic acid. Local anesthetic (ropivacaine) was administered intraoperatively and continued as needed for postoperative analgesia.

The patient was discharged on postoperative day six (December 24, 2024) on oral antibiotics (amoxicillin‐clavulanic acid 375 mg three times daily for 4 days) and analgesics (paracetamol as needed).

### 2.7 Histopathological Findings

Histopathological examination (On January 9, 2025) of the excised retroperitoneal mass revealed a partially encapsulated specimen measuring 12 × 10 × 10 cm, which was already bihalved. The outer surface was irregular and encapsulated. On cut section, the cyst wall thickness ranged from 0.2 to 0.4 cm, with a solid Rokitansky protuberance measuring 7 × 7 × 3 cm. The contents included cheesy pultaceous material, hair, teeth, bone, and yellow fatty areas.

**FIGURE 3:**
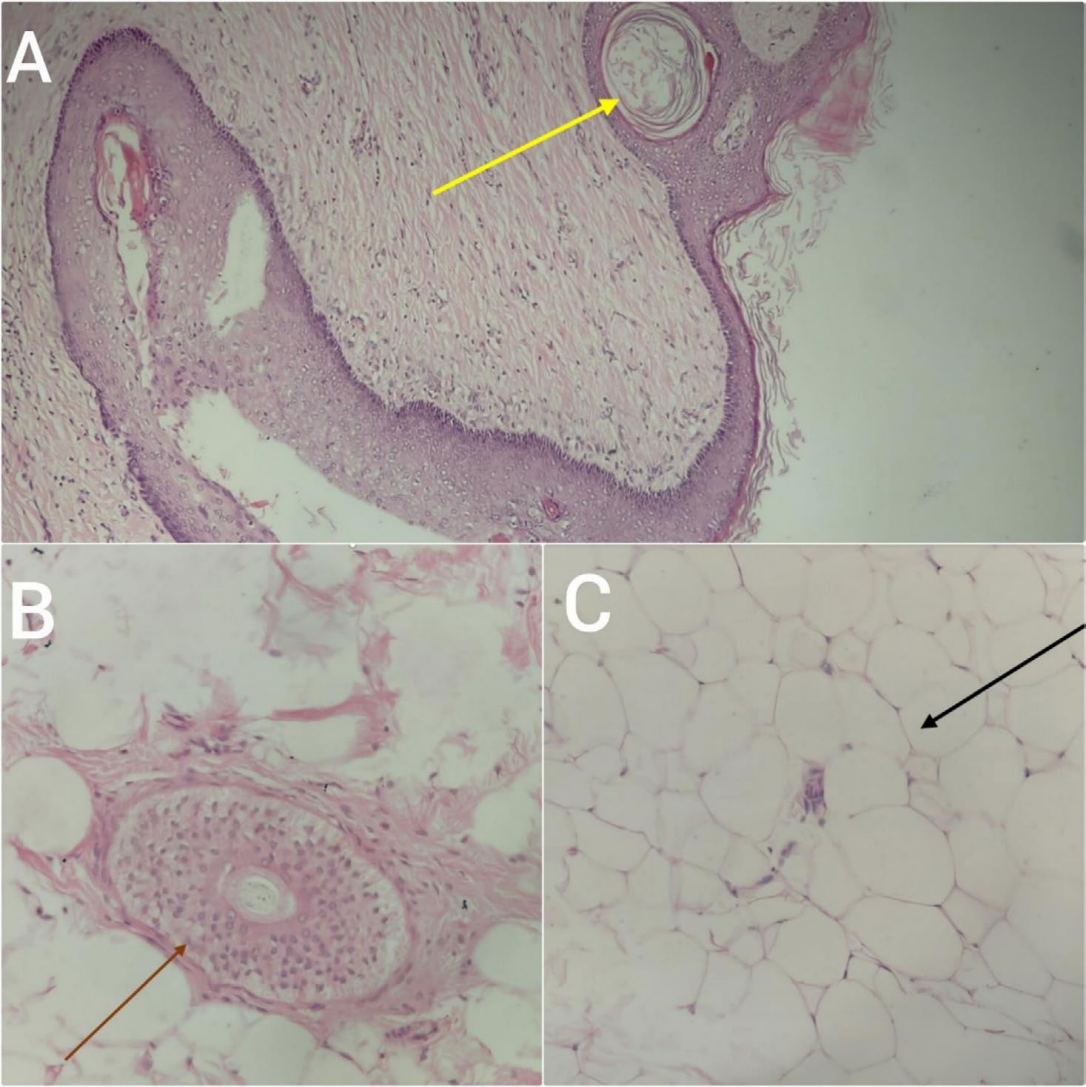
Histopathological features of the retroperitoneal mature cystic teratoma (H&E stain ×10 and ×40). (A) Keratinized stratified squamous epithelium (keratin plug) with a hair follicle (yellow arrow). (B) Cross‐section of a hair follicle (brown arrow) embedded in connective tissue. (C) Adipose tissue with compressed adrenal cortical cells (black arrow). ×10 magnification for Figure 3A and ×40 magnification for Figure 3B,C.

Microscopic evaluation of hematoxylin and eosin (H&E)‐stained sections (×10 and ×40) demonstrated tissues derived from all three germ layers, consistent with a teratoma. The tissue is lined by keratinized stratified squamous epithelium with underlying adnexal structures, including pilosebaceous glands, hair follicles, and hair shafts (Figure 3A (×10) and 3B (×40)). Areas lined by respiratory‐type pseudostratified columnar epithelium and intestinal epithelium were also identified. Mesodermal elements included areas of calcification, cartilage, adipose tissue, and smooth muscle. Nerve bundles were noted adjacent to the intestinal lining, along with mature neural components. A foreign body reaction was evident in focal areas, characterized by multinucleated giant cells, dense chronic inflammatory infiltrates, and cholesterol clefts. On 40×, compressed normal adrenal tissue was present, comprising cells of the zona fasciculata with clear vacuolated cytoplasm arranged in cords and fascicles, surrounded by areas of hemorrhage Figure 3C (×40). Multiple sections were examined, and no immature components or features of malignancy were observed.

Immunohistochemistry was not performed as it was deemed unnecessary for confirming the diagnosis of mature teratoma.

Decalcified bony tissue sections revealed multiple tissue types, including bony tissue with marrow cavities containing hematopoietic cells, areas of endochondral ossification, cartilage, smooth muscle, and fibroadipose tissue. No evidence of immature components or malignancy was identified.

The final histopathological diagnosis was mature cystic teratoma of the retroperitoneum, negative for malignancy.

### 2.8 Follow‐Up and Complications

The patient developed wound dehiscence with serous discharge noted during follow‐up on December 30, 2024, possibly due to wound tension and nutritional factors. Despite regular dressing and a high‐protein diet, the discharge persisted, necessitating secondary suturing on January 12, 2025. No imaging was performed to exclude intra‐abdominal collection as clinical signs were absent. Sutures were removed on January 23, 2025, with satisfactory wound healing.

At the six‐month follow‐up (July 13, 2025), the patient presented with a hypertrophic scar at the incision site without discharge or constitutional symptoms. The scar was managed with intralesional triamcinolone injection with plans for serial injections if patient party complies. Postoperative tumor markers (AFP) remained normal, and surveillance ultrasonography showed no evidence of recurrence; however, longer‐term imaging is planned. (Table 1.) provides the timeline of the case till follow up.

**TABLE 1:** Summary of the timeline of key clinical events.

Date	Event
October 23, 2024	Ultrasonography at peripheral hospital reveals right suprarenal mass; referred to our center.
October 24, 2024	CECT abdomen and pelvis confirms heterogeneous suprarenal lesion.
October–November 2024	Conservative management due to financial constraints; regular follow‐ups.
December 19, 2024	Exploratory laparotomy and complete excision of mass.
December 24, 2024	Discharged on postoperative day 6.
December 30, 2024	Wound dehiscence noted; managed with dressings.
January 9, 2025	Histopathology confirms mature teratoma.
January 12, 2025	Secondary suturing for dehiscence.
January 23, 2025	Sutures removed; wound healed.
July 13, 2025	Six‐month follow‐up: Asymptomatic, no recurrence; hypertrophic scar managed.

## 3 Discussion

Primary adrenal teratomas represent an exceptionally rare entity in pediatric surgery, with our case adding to the limited body of literature on this condition. The clinical presentation, imaging characteristics, and successful surgical management of this case provide valuable insights into the diagnosis and treatment of these uncommon tumors.

The pathogenesis of adrenal teratomas remains unclear. The most widely accepted theory suggests aberrant migration of primordial germ cells along the urogenital ridge during embryonic development, favored due to the presence of tissues from all three germ layers and the tumor's midline location; this is corroborated by recent antenatal case reports [2, 13, 14]. Alternative hypotheses include metaplastic transformation of adrenal cortical cells or development from embryonic cell rests displaced during organogenesis [15]. The presence of all three germ layers in our case supports the germ cell origin theory.

Clinical presentation of adrenal teratomas is typically nonspecific, as observed in our patient who presented with intermittent abdominal pain. A review of pediatric case report and literature reveals that most patients present between 3–15 years of age, with a slight female predominance, consistent with 2024 reports of similar demographics in infants and children [5, 16, 17]. For instance, our 7‐year‐old female patient with a right‐sided 11 × 8 cm mass aligns with reported cases where mean age is around 5–10 years, right side predominance, and sizes ranging from 5–15 cm, with favorable outcomes post‐excision [14, 16, 17]. Unlike functional adrenal tumors, teratomas rarely produce hormones, and biochemical markers including catecholamines, cortisol, and androgens are typically normal [12, 18]. Our patient's normal AFP levels and absence of endocrine symptoms were consistent with a non‐functional mature teratoma.

Imaging plays a crucial role in the preoperative evaluation of adrenal masses. The characteristic imaging features of teratomas include a heterogeneous mass with fat, fluid, and calcific components [19]. The presence of fat‐fluid levels, as seen in our case, is highly suggestive of teratoma, though not pathognomonic, and recent imaging in pediatric cases reinforces the utility of CECT for diagnosis [14, 20]. The differential diagnosis of a calcified suprarenal mass in children includes neuroblastoma (the most common), ganglioneuroma, adrenocortical carcinoma, adrenal hematoma, and pheochromocytoma; antenatal cases often mimic neuroblastoma on initial imaging [14, 21]. Neuroblastoma typically shows elevated urinary catecholamines and may cross the midline, features absent in our case [22]. The complementary role of imaging (e.g., fat‐fluid levels on CECT) and tumor markers (normal AFP, catecholamines) is essential for narrowing the differential.

CECT remains the imaging modality of choice for evaluating retroperitoneal masses, providing excellent anatomical detail and helping assess resectability [23]. The presence of fat density within the mass strongly suggests teratoma, though lipomatous components can occasionally be seen in other retroperitoneal tumors [24]. Magnetic resonance imaging may provide additional soft tissue characterization but was not performed in our case due to financial constraints.

Surgical excision remains the definitive treatment for adrenal teratomas, serving both therapeutic and diagnostic purposes [25]. Complete excision is crucial to prevent potential complications including malignant transformation, rupture, infection, or mass effect on adjacent structures [26]. The surgical approach depends on tumor size, location, and surgeon preference. While laparoscopic adrenalectomy has been increasingly reported for smaller tumors, open surgery remains the standard for large masses with adhesions to vital structures; this approach was successful in recent giant pediatric teratoma excisions [16, 27]. In resource‐limited settings like ours, challenges include delayed diagnosis due to financial barriers and limited access to Pediatric laparoscopy apparatus, leading to open approaches.

The intraoperative finding of dense adhesions to the inferior vena cava in our case necessitated meticulous dissection to achieve complete excision while preserving vascular integrity—a key surgical pearl to avoid vascular injury. The presence of multiple feeding vessels, as encountered in our case, requires careful ligation to minimize blood loss [28]. Despite the tumor's large size and adhesions, complete excision was achieved without major complications, emphasizing the importance of avoiding rupture during dissection.

Histopathological examination is essential for definitive diagnosis and to exclude malignant elements. Mature teratomas show well‐differentiated tissues from all three germ layers without immature components; recent histopathological analyses in pediatric adrenal cases confirm the absence of malignancy in most instances [16, 17, 29]. The presence of immature elements, found in approximately 20% of cases, does not necessarily indicate malignancy in prepubertal children but requires close follow‐up [30]. Malignant transformation, though rare in pediatric cases, has been reported and emphasizes the importance of complete excision and long‐term surveillance [9].

The postoperative complication of wound dehiscence in our patient, while not directly related to the tumor pathology, highlights the importance of meticulous wound care and nutritional support in pediatric patients undergoing major abdominal surgery. The development of hypertrophic scarring, managed with intralesional corticosteroids, is a recognized complication following laparotomy in children [31].

Long‐term prognosis following complete excision of benign adrenal teratomas is excellent, with recurrence being exceptionally rare; follow‐up in recent cases supports this with no recurrences reported at 6–12 months [17, 32]. Regular follow‐up with imaging is recommended, particularly in the first 2 years post‐surgery, to detect any recurrence or metachronous tumors, with emphasis on longer surveillance given the potential for late malignant transformation [33]. Our patient remains disease‐free at 6 months follow‐up, though longer surveillance is warranted.

## 4 Limitations

Several limitations need acknowledgment. Preoperative biopsy was not indicated due to the risk of tumor seeding and diagnostic uncertainty. No surgical resection pictures are available. At submission, only six‐month follow‐up data is available, which is short; longer imaging surveillance is recommended to detect potential recurrence or malignant transformation.

## 5 Conclusion

Primary adrenal teratomas, though very rare in the pediatric population, should be considered in the differential diagnosis of suprarenal masses, particularly when imaging demonstrates fat, fluid, and calcific components. Complete surgical excision remains the treatment of choice, providing both definitive diagnosis and cure. Despite the technical challenges posed by large tumors with vascular adhesions, careful surgical technique can achieve complete excision with minimal morbidity. Long‐term prognosis following complete excision of mature teratomas is excellent, though regular surveillance is recommended. This case adds to the limited literature on pediatric adrenal teratomas and emphasizes the importance of maintaining a high index of suspicion for this rare entity in children presenting with suprarenal masses, especially in resource‐limited settings where early intervention may be delayed.

## Author Contributions

**Amit Gautam:** conceptualization, investigation, methodology, project administration, supervision, writing – original draft, writing – review and editing. **Pukar Adhikari:** conceptualization, data curation, investigation, methodology, project administration, visualization, writing – original draft, writing – review and editing. **Shishir Bhandari:** data curation, validation, writing – original draft, writing – review and editing. **Jabir Ahamad Miya:** data curation, validation, writing – original draft, writing – review and editing. **Nikesh Tiwari:** data curation, writing – original draft, writing – review and editing.

## Funding

The authors have nothing to report.

## Ethics Statement

Ethical approval was not required for this single case report.

## Consent

Written informed consent was obtained from the patient's parents for publication of this case report and accompanying images.

## Conflicts of Interest

The authors declare no conflicts of interest.

## Data Availability

Supporting data are available from the corresponding author upon reasonable request, subject to privacy and ethical restrictions.
